# Analysis of middle-aged and older adults’ willingness concerning living wills and associated factors

**DOI:** 10.3389/fpubh.2025.1743077

**Published:** 2026-01-13

**Authors:** Linyu Jiang, Yuwan Duan, Daping Li

**Affiliations:** School of Public Health, Guangdong Medical University, Dongguan, Guangdong, China

**Keywords:** associated factors, cross-sectional study, Firth’s penalized logistic regression analysis, living will, willingness

## Abstract

**Objective:**

To investigate the current status of Living Will willingness among middle-aged and older adults in Shenzhen, China, and to systematically identify its associated influencing factors.

**Methods:**

Based on the Theory of Planned Behavior and the Life Course Theory, a cross-sectional survey was conducted from May 2023 to February 2024. A total of 519 middle-aged and older adults were recruited in Shenzhen. Data were collected using a general information questionnaire, a Living Will knowledge questionnaire, a Living Will attitude scale, a family APGAR scale, a health status scale, and a social support scale. Chi-square test, Mann–Whitney U test, and Firth penalized logistic regression analysis were employed to identify associated factors.

**Results:**

The proportion of participants with a positive willingness toward creating a Living Will was 64.7%. Significant influencing factors included age (*p* = 0.033), being a healthcare professional (*p* = 0.033), being a civil servant or employee of a public institution/enterprise (*p* = 0.033), experience of witnessing a rescue or death (*p* = 0.033), knowledge score (*p* = 0.032), attitude score (*p* = 0.007), family function (*p* = 0.003), subjective social support (*p* = 0.009), and utilization of social support (*p* = 0.027). Furthermore, an educational level of college or above (*p* = 0.053), monthly income (*p* = 0.067), religious belief (*p* = 0.073), physical health status (*p* = 0.073), psychological health status (*p* = 0.099), and objective social support (*p* = 0.055) showed borderline significance.

**Conclusion:**

Middle-aged and older adults in Shenzhen demonstrated a relatively positive willingness regarding Living Wills. Nine protective factors were identified, including a higher education level, presence of religious belief, higher monthly income, experience of witnessing medical rescue or death, better knowledge of Living Wills, a positive attitude toward Living Wills, good family function and psychological status, and strong social support.

## 1 Introduction

With the continuous deepening of population aging and the rising prevalence of chronic diseases, the dilemmas in medical decision-making faced by end-of-life patients have become increasingly prominent. The traditional medical model, which is predominantly oriented toward life-prolonging, can no longer adequately meet patients’ diverse and high-level needs, such as alleviating suffering and preserving dignity. The Living Will system, grounded in the principle of patient autonomy and the principle of non-maleficence in modern medical ethics, has thus emerged. A Living Will is a written document through which an individual with full civil capacity, while mentally lucid, expresses in advance their wishes to accept or refuse specific medical interventions (such as cardiopulmonary resuscitation, endotracheal intubation, etc.) should they become terminally ill or fall into an irreversible coma (1). The guiding principles of the Living Will are to respect individual autonomy, avoid excessive medical treatment, enhance the quality of life, and alleviate the economic and emotional burdens on families (2). Within China’s current legal system, Shenzhen has taken a pioneering step by granting Living Wills explicit legal validity through local legislation. The Shenzhen Special Economic Zone Medical Regulations (2023) stipulate that when a patient or their close relatives provide a qualified Living Will, medical institutions must respect the patient’s directives stated therein when implementing medical measures during an incurable terminal stage of an illness or at the end of life (3). With the legal validation of Living Wills in Shenzhen and the implementation of a series of supporting policies, systematically investigating the willingness and identifying influencing factors within specific pilot areas where Living Wills have gained legal recognition holds significant theoretical value and practical importance for promoting the localized adaptation and development of the Living Will system. It also carries relevant implications for civil affairs, justice, and health administration. Therefore, in this study, willingness regarding Living Wills is defined as the intention to create a Living Will, aiming to understand this willingness and its associated factors among adults aged 45 years and older in Shenzhen.

The Theory of Planned Behavior explains that the formation of a behavior involves cognition, attitudes, and subjective norms, emphasizing the thought and action processes individuals undertake when making plans and executing behaviors (4). Applying this theoretical framework, the study delves into how individuals’ attitudes toward Living Wills, their perceived social resources, and their self-assessed capacity to execute such decisions collectively shape their willingness. The Life Course Theory posits that an individual’s life trajectory is shaped not by isolated factors, but through continuous interactions between the individual and their socio-historical environment. This influence stems from both macro-level social structures and micro-level personal choices and practices, affecting Living Will willingness across the dimensions of time (age, generation), level (psychological and physiological traits, socioeconomic status, cultural context), and domain (various life spheres such as family, occupation, and education) (5). Guided by the Theory of Planned Behavior and the Life Course Theory, this study investigated the willingness toward Living Wills among middle-aged and older adults using a general information questionnaire, a Living Will knowledge questionnaire, a Living Will attitude scale, a family APGAR scale, a health status scale, and a social support scale. The findings aim to provide evidence for adjusting Living Will policies and promote the optimization of these policies and the refinement of their practical implementation.

## 2 Materials and methods

### 2.1 Study participants

This study was approved by the Clinical Research Ethics Committee of the Affiliated Hospital of Guangdong Medical University (Approval No. PJKT2024-001). From May 2023 to February 2024, based on the gender and education level statistics from the Shenzhen population census, a quota sampling method was employed. Participants were recruited from community health service stations, general hospitals, and street committees in densely populated districts of Shenzhen, including Nanshan, Longgang, Longhua, and Futian, using a combination of on-site recruitment and online invitations.

**Figure 1:**
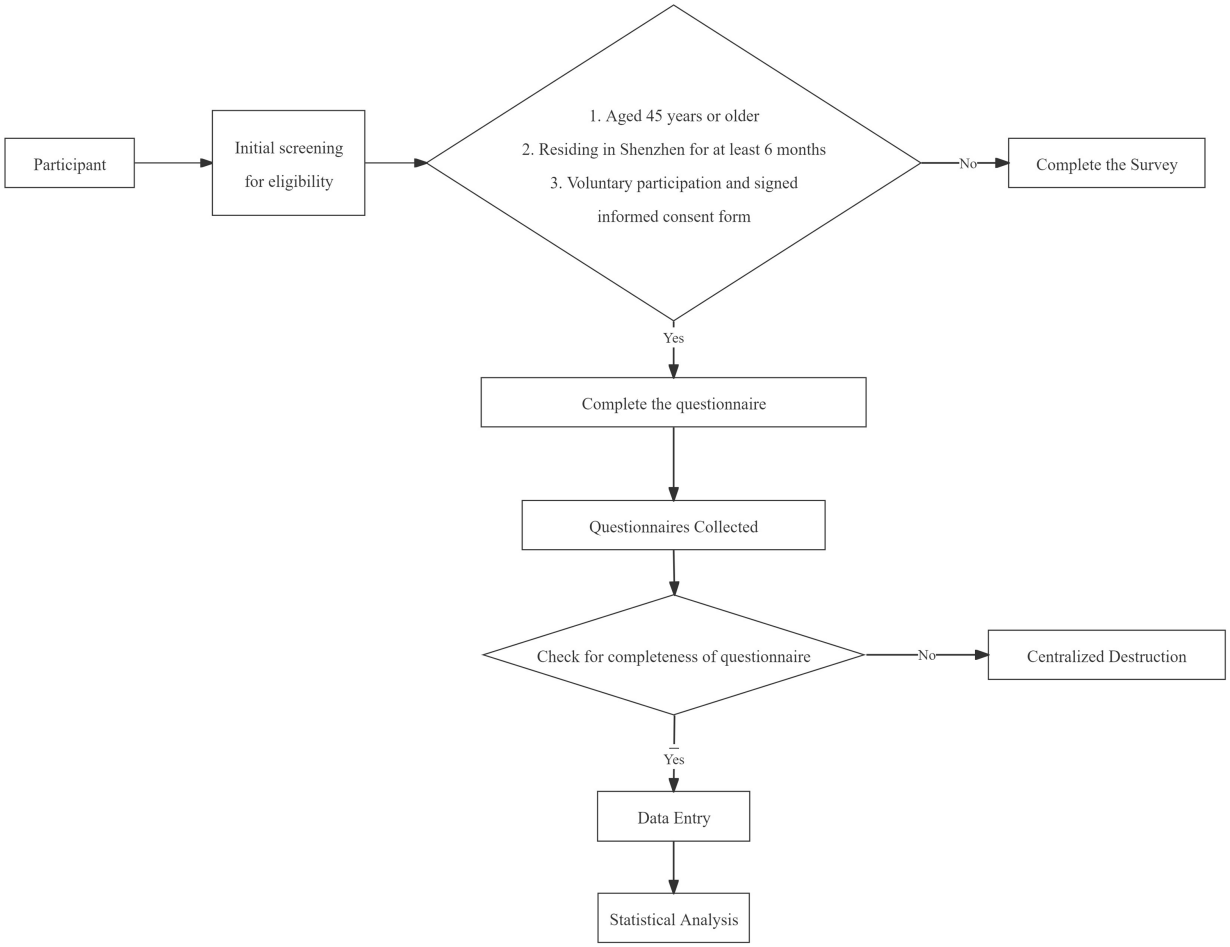
Flowchart of participant recruitment and living will questionnaire collection.

The inclusion criteria were: middle-aged and older adults aged 45 years or above, who had been permanent residents of Shenzhen for at least 6 months, and who voluntarily participated and provided signed informed consent. The exclusion criteria were: individuals with cognitive impairment or those unable to complete the questionnaire independently due to severe illness, and those who declined to participate. The sample size was calculated using the formula: Based on a small-scale pilot survey indicating an expected agreement rate of 53.5% regarding willingness toward Living Wills among the target population, and setting *α* at 0.05, the calculated minimum required sample size was approximately 382. A total of 550 questionnaires were distributed and returned. Among these, 519 were valid, resulting in an effective response rate of 94.4%. The participant flow diagram is presented in Figure 1.

### 2.2 Methods

#### 2.2.1 General information questionnaire

The questionnaire was self-developed by the research team based on a systematic literature review. It covered the following variables: age, gender, occupation (including pre-retirement occupation), monthly income, educational attainment, number of children raised, religious belief, and whether the individual had witnessed medical rescue or the death of a dying person.

#### 2.2.2 Living will knowledge

A Living Will knowledge questionnaire was developed based on previous studies (6, 7), the provisions regarding the content and implementation of Living Wills outlined in the Shenzhen Special Economic Zone Medical Regulations (3), and in-depth interviews with the target population. The questionnaire comprises five items covering the concept of Living Wills, their specific content, the methods for formalizing a Living Will, personal values, and the right to know about one’s medical condition. A Likert 5-point scale was employed for scoring, ranging from 1 (indicating complete lack of understanding) to 5 (indicating thorough understanding). Higher scores reflect a more sufficient level of knowledge about Living Wills among the respondents. A pilot test indicated that the Cronbach’s *α* coefficient for the Living Will knowledge items was 0.85.

#### 2.2.3 Assessment of attitudes toward living wills

Attitudes toward Living Wills were measured using the “Living Will Attitude Scale” (8), which was originally developed by Akabayashi and introduced into the Chinese context by Zhang Qiu through translation. The scale consists of 7 items, each rated on a 5-point Likert scale ranging from 1 (strongly disagree) to 5 (strongly agree). The total score, calculated as the sum of all item scores, represents the final attitude toward Living Wills, with higher scores indicating a more positive attitude. A pilot test demonstrated that the scale had excellent internal consistency, with Cronbach’s *α* coefficient of 0.95.

#### 2.2.4 The family APGAR scale

The Family APGAR Scale, revised by Meng (9), was employed to measure participants’ subjective satisfaction with family function. The scale comprises five items assessing family adaptation, partnership, growth, affection, and resolve. Each item is rated on a 3-point scale, ranging from 2 (“always like this”) to 0 (“hardly ever”). Higher total scores indicate a greater perceived level of family support. A pilot test demonstrated that the scale had a Cronbach’s *α* coefficient of 0.87.

#### 2.2.5 Short form-8 health survey

The health status of middle-aged and older adults was assessed using the universally employed Short Form-8 Health Survey (SF-8) (10). This scale comprises 8 items evaluating both physical and mental health summaries. Item 4 utilizes a 6-point rating system, while the remaining items employ a 5-point rating method. The health status scores are derived from weighted integration of the item scores, with higher scores indicating better health status. A pilot test demonstrated that the Cronbach’s *α* coefficient for the health status scale was 0.87.

#### 2.2.6 Social Support Rating Scale

Social support was assessed using the “Social Support Rating Scale” (SSRS) developed by Xiao Shuiyuan based on the Chinese social context (11) This scale comprises three dimensions: objective support, subjective support, and utilization of support For the scoring method: Items 1–4 and 8–10 are single-choice; selections 1–4 are scored 1–4 points, respectively. Item 5 is calculated as the sum of its four sub-items, each scored from 1 (“none”) to 4 (“full support”). For Items 6 and 7, a response indicating “no source” scores 0 points, while choosing “the following sources” scores 1 point for each source selected. Higher total scores indicate a better level of social support. A pilot test showed that the Cronbach’s *α* coefficient for the Social Support Rating Scale was 0.78.

### 2.3 Statistical analysis

The survey data were double-entered and managed using EpiData software (version 3.1). Outliers were identified using the boxplot method. Each identified outlier was individually assessed in conjunction with the clinical context: those confirmed as recording errors were excluded, while physiologically extreme true values were retained. Data consistency was checked to ensure quality. Statistical analysis and data organization were performed using SPSS (version 25.0).

Categorical data are presented as frequency (percentage) and were compared using the Chi-square test. The Kolmogorov–Smirnov test indicated that all continuous variables deviated from a normal distribution (*p* < 0.05); therefore, they are expressed as Median (Interquartile Range, IQR) and compared using the Mann–Whitney U test.

Univariate analysis was used to screen for significant factors. After testing for multicollinearity, variables including unordered demographic variables and composite scores from six measurement dimensions (which were significant in the univariate analysis)—Living Will knowledge, attitude toward Living Wills, family function, physical health summary, mental health summary, and objective support—were included as independent variables in a Firth penalized logistic regression model. A two-sided significance level of *α* = 0.05 was set for all tests.

## 3 Results

### 3.1 Baseline characteristics of the study subjects

Individuals aged 45–59 years constituted 69.2% of the study sample. The proportion of middle-aged and older adults with a positive willingness toward Living Wills was 64.7% (95% CI: 0.606–0.688). Regarding educational attainment, 49.1% of those with a junior high school education and 78.3% of those with a college degree or above expressed a positive willingness. Concerning occupation, 75.3% of healthcare professionals reported a positive willingness. In terms of monthly income, 62.7% of individuals with an income between 2,000 and 5,000 RMB, and 82.2% of those with an income of 10,000 RMB or more, demonstrated a positive willingness. Among those with religious beliefs, 72.3% showed a positive willingness. Notably, 82.1% of those who had witnessed a medical rescue or a death exhibited a positive willingness. Statistical analysis revealed that variables including educational level, occupation type, monthly income, number of children raised, religious belief, and experience of witnessing a medical rescue or death showed statistically significant differences (*p* < 0.05) between groups with different levels of willingness toward Living Wills (see Table 1 for details).

**Table 1:** Baseline characteristics of the study subjects (*N* = 519).

Variable	Options	Willingness to complete a living will		*χ* ^2^	*Ρ*
		No (183 individuals)	Yes (336 individuals)		
Gender	Male	80(35.6%)	145(64.4%)	0.015	0.902
	Female	103(35.0%)	191(65.0%)		
Age (years)	45–59	126(35.1%)	233(64.9%)	0.684	0.710
	60–69	44(34.1%)	85(65.9%)		
	70 and above	13(41.9%)	18(58.1%)		
Educational level	Primary school or below	33(46.5%)	38(53.5%)	29.629	<0.001
	Junior high school	55(50.9%)	53(49.1%)		
	High school or secondary specialized school	57(34.5%)	108(65.5%)		
	College degree or above	38(21.7%)	137(78.3%)		
Occupation	Production and related technical workers	37(41.1%)	53(58.9%)	12.734	0.047
	Healthcare professionals	19(24.7%)	58(75.3%)		
	Government or public institution employees	33(35.9%)	59(64.1%)		
	Company employees	41(29.1%)	100(70.9%)		
	Police and firefighters	8(42.1%)	11(57.9%)		
	Agriculture, forestry, animal husbandry, fishery, and water conservancy workers	7(36.8%)	12(63.2%)		
	Unemployed	38(46.9%)	43(53.1%)		
Monthly Income (RMB)	[0–2,000)	55(64.7%)	30(35.3%)	50.180	<0.001
	[2,000–5,000)	63(37.3%)	106(62.7%)		
	[5,000–10,000)	44(29.9%)	103(70.1%)		
	≥10,000	21(17.8%)	97(82.2%)		
Number of children raised	0	21(67.7%)	10(32.3%)	19.803	<0.001
	1	76(30.3%)	175(69.7%)		
	2	61(33.5%)	121(66.5%)		
	3 or more	25(45.5%)	30(54.5%)		
Religious belief	No	122(40.8%)	177(59.2%)	9.493	0.002
	Yes	61(27.7%)	159(72.3%)		
Witnessed medical rescue or end-of-life death	No	133(55.6%)	106(44.4%)	80.671	<0.001
	Yes	50(17.9%)	230(82.1%)		

### 3.2 Univariate analysis of measurement dimensions

Among the study participants with a positive willingness toward Living Wills, the median scores (with interquartile range, IQR) were as follows: Living Will knowledge score 15 (5), attitude score 28 (7), family APGAR score 5 (4), physical component summary score 34.0 (90), mental component summary score 300 (125), and objective social support score 8 (5).

With the willingness toward Living Wills among middle-aged and older adults as the dependent variable, univariate analysis for continuous independent variables that did not conform to a normal distribution was performed using the nonparametric Mann–Whitney U test. Among the scale dimensions, six dimensions—Living Will knowledge, attitude toward Living Wills, family function, physical component summary, mental component summary, and objective support—showed statistically significant differences (*p* < 0.05) in relation to Living Will willingness (see Table 2 for details).

**Table 2:** Univariate analysis of factors associated with the willingness toward living wills among middle-aged and older adults

Dimensions.	Willingness to complete a living will median (IQR)		*Ζ*	*Ρ*
	No	Yes		
Living will knowledge	13(10,15)	15(13,18)	−6.917	<0.001
Attitude toward living wills	21(18,28)	28(25,32)	−9.099	<0.001
Family support	5(3,7)	5(5,9)	−3.605	<0.001
Physical component summary	280(225,355)	340(290,380)	−5.686	<0.001
Mental component summary	250(200,325)	300(250,375)	−4.765	<0.001
Subjective support	22(18,25)	23(19,26)	−1.709	0.087
Objective support	4(3,7)	8(5,10)	−9.237	<0.001
Utilization of support	6(5,8)	7(6,8)	−1.909	0.056

### 3.3 Firth penalized logistic regression analysis of factors associated with willingness toward living wills

Collinearity diagnostics indicated that the variance inflation factor (VIF) for each independent variable was less than 3, suggesting no evidence of multicollinearity. Using the willingness toward a Living Will (no = 0, yes = 1) as the dependent variable, a Firth penalized logistic regression model was constructed with the following independent variables: educational level, religious belief, occupation, monthly income, experience of witnessing medical rescue or death, number of children raised, and the composite scores of Living Will knowledge, attitude toward Living Wills, family function, physical component summary, mental component summary, and social support. The model demonstrated a good overall fit (*χ*^2^ = 85.34, *p* < 0.001), with a Nagelkerke R^2^ of 0.428, indicating that 42.8% of the variance was explained. The results revealed that significant influencing factors included age (*p* = 0.033), being a healthcare professional (*p* = 0.033), being a civil servant or employee of a public institution/enterprise (*p* = 0.033), experience of witnessing medical rescue or death (*p* = 0.033), knowledge score (*p* = 0.032), attitude score (*p* = 0.007), family function (*p* = 0.003), subjective support (*p* = 0.009), and utilization of support (*p* = 0.027). Furthermore, an educational level of college or above (*p* = 0.053), monthly income (*p* = 0.067), religious belief (*p* = 0.073), physical component summary (*p* = 0.073), mental component summary (*p* = 0.099), and objective support (*p* = 0.055) showed a trend toward significance (borderline significance) (see Table 3 for details).

**Table 3:** Results of Firth’s penalized logistic regression analysis of factors associated with the willingness toward living wills among middle-aged and older adults.

Variables	Beta	SE	*Z*	*P*	OR (95% CI)
Demographic characteristics					
Age	0.032	0.015	2.13	0.033	1.033(1.003–1.064)
Gender	−0.245	0.187	−1.31	0.190	0.783(0.543–1.129)
Marital status	0.128	0.094	1.36	0.174	1.137(0.945–1.368)
Educational level (reference group: primary school)					
Junior high school vs. primary school	0.215	0.237	0.91	0.364	1.240(0.780–1.973)
High school/secondary specialized school vs. Primary school	0.342	0.251	1.36	0.173	1.408(0.861–2.304)
College degree or above vs. primary school	0.518	0.268	1.93	0.053	1.678(0.992–2.839)
Occupation					
Healthcare professionals	0.892	0.312	2.86	0.004	2.440(1.324–4.498)
Government or public institution employees	0.634	0.285	2.22	0.026	1.886(1.079–3.298)
Company employees	0.428	0.271	1.58	0.114	1.534(0.902–2.610)
Monthly Income	0.189	0.103	1.83	0.067	1.208(0.987–1.479)
Number of children raised	0.056	0.089	0.63	0.529	1.058(0.888–1.260)
Religious belief (yes vs. no)	0.312	0.174	1.79	0.073	1.366(0.972–1.920)
Experience of witnessing medical rescue or end-of-life death (yes vs. no)	0.467	0.192	2.43	0.015	1.595(1.095–2.323)
Living will knowledge score	0.045	0.021	2.14	0.032	1.046(1.004–1.090)
Attitude toward living wills score	0.067	0.025	2.68	0.007	1.069(1.018–1.123)
Family APGAR (family function) score	0.123	0.042	2.93	0.003	1.131(1.042–1.228)
Physical component summary (SF-8) score	−0.034	0.019	−1.79	0.073	0.967(0.931–1.004)
Mental component summary (SF-8) score	0.028	0.017	1.65	0.099	1.028(0.995–1.063)
Subjective support score	0.039	0.015	2.60	0.009	1.040(1.010–1.071)
Objective support score	0.025	0.013	1.92	0.055	1.025(0.999–1.052)
Utilization of support score	0.031	0.014	2.21	0.027	1.031(1.003–1.060)

## 4 Discussion

### 4.1 The crucial enabling role of legal support in enhancing the willingness to establish a living will

Middle-aged and older adults’ understanding of the legal knowledge related to creating a Living Will is positively correlated with the positivity of their willingness. The Shenzhen Special Economic Zone Medical Regulations (2023) (3) specify the eligibility for signing a Living Will (individuals with full civil capacity), the conditions for its validity (made while mentally lucid), and its core content (the scope of medical measures that can be refused or accepted). In this study, the 64.7% rate of positive willingness is significantly associated with the cognition of Living Wills among middle-aged and older adults in Shenzhen—in contrast to non-pilot areas in China, where the lack of clear legislation and weak public legal knowledge result in a lower proportion of positive willingness (12). This also demonstrates that the sense of trust fostered by legal safeguards can further strengthen willingness.

### 4.2 Experiences, religious belief, economic status, and educational level are positively associated with middle-aged and older adults’ willingness toward living wills

For individuals with religious beliefs, the coefficient was 0.312, with an odds ratio (*OR*) of 1.366 (95% CI: 0.972–1.920; *p* = 0.073), indicating a higher willingness toward Living Wills, albeit with borderline significance. Among middle-aged and older adults who had witnessed medical rescue or end-of-life death, the coefficient for Living Will willingness was 0.467, corresponding to an *OR* of 1.595 (95% CI: 1.095–2.323; *p* = 0.015), demonstrating a significantly higher willingness among those with such experiences. Individuals with such experiences may inherently exhibit higher death anxiety, stronger self-reflection on life, or greater access to medical information channels, thereby enhancing their willingness toward Living Wills. For monthly income, the coefficient was 0.189, with an *OR* of 1.208 (95% CI: 0.987–1.479; *p* = 0.067), suggesting a positive influence on willingness, though marginally significant. Saeed (13) also confirmed in previous studies that economic factors play a significant role in shaping Living Will decisions among middle-aged and older adults. These findings reveal that individuals with higher economic income levels possess greater accessibility to high-quality healthcare services and are more likely to acquire the legal support and medical knowledge resources necessary for creating a Living Will. For instance, regular health check-ups or health management interactions not only increase the likelihood of understanding Living Wills but also provide opportunities to discuss health maintenance with healthcare professionals, reinforcing the concept of “proactive health planning” and enhancing the overall quality of life.

The study found that higher educational attainment was associated with a more positive willingness toward creating a Living Will. Groups with higher levels of education demonstrated distinct advantages in having diverse channels for information acquisition and a stronger capacity for critically interpreting media content. This enabled them to proactively seek out and effectively discern professional information related to Living Wills. Such a structural advantage in information resources significantly enhanced their depth of understanding and willingness to accept the Living Will system. For the group with an educational level of college degree or above, the coefficient was 0.518, with an odds ratio (*OR*) of 1.678 (95% CI: 0.992–2.839; *p* = 0.053). Compared to the group with a primary school education level, those with a college degree or above showed a higher willingness, which was marginally significant (*p* = 0.053). This data clearly underscores that “knowledge is the foundation, attitude is the key”—indicating that the current societal penetration of knowledge about Living Wills remains insufficient, while a positive attitude can more powerfully drive the transformation of willingness from the “cognitive level” to the “acceptance level.” This finding further highlights the crucial role of a positive attitude in facilitating and strengthening the formation of willingness toward Living Wills. In this study sample, middle-aged individuals aged 45–59 accounted for a high proportion of 69.2%. This age group is typically in the special life stage of the “sandwich generation,” simultaneously bearing responsibilities for parental care and child-rearing, while also beginning to face potential threats to their own health. As they gradually approach the transition point of their career conclusion, this period is more likely to provoke deep reflection on the finitude of life and arrangements for the end-of-life, leading to a relatively higher level of awareness and concern regarding relevant Living Will policies and regulations, which consequently contributes to the formation of a higher level of willingness toward Living Wills.

### 4.3 The health status was negatively associated, while family care and social support were positively associated with the older adults’ willingness to establish a Living Will

#### 4.3.1 Middle-aged and older adults in poorer health demonstrated an increased level of willingness toward creating a living will

Better self-rated health status among middle-aged and older adults was associated with a lower willingness toward creating a Living Will. For the physical component summary score, the coefficient was −0.034, with an odds ratio (*OR*) of 0.967 (95% CI: 0.931–1.004; *p* = 0.073), indicating a marginally significant negative association (*p* = 0.073). This finding contrasts with the results reported in previous studies by Hirakawa (14) and Youn (15). The willingness to complete a Living Will essentially represents a form of prospective medical decision-making planning. Individuals in good health may be more likely to overlook the importance of proactively planning for future healthcare scenarios. This pattern also suggests that the onset of health problems can serve as a catalyst, prompting people to contemplate their own medical planning. However, it is crucial to correct the common misconception that only those who are seriously ill need to establish a Living Will. In fact, individuals who are clear-minded, possess full decision-making capacity, and can autonomously express their true wishes are ideally positioned to be the primary target group for Living Will promotion. Encouraging these individuals to complete a Living Will in a timely manner can effectively avoid missing the optimal window for creating one, which might otherwise be lost due to “declined cognitive capacity in the advanced stages of an illness” or “psychological resistance when facing a severe diagnosis.”

#### 4.3.2 Middle-aged and older adults with a higher level of family support demonstrate a greater willingness to establish a living will

Middle-aged and older adults with higher levels of family function demonstrated a greater willingness toward creating a Living Will. The Firth penalized logistic regression model indicated a coefficient of 0.123 for family function, with an odds ratio (*OR*) of 1.131 (95% CI: 1.042–1.228; *p* = 0.003). For each one-unit increase in family function, the odds of expressing willingness increased by 13.1%, indicating a statistically significant effect. Middle-aged and older adults who received more adequate support and care within the family system exhibited a significantly higher level of willingness toward Living Wills and were more likely to complete one, aligning with the findings reported by Glaudemans (16) and Stone (17). This phenomenon is deeply rooted in the traditional Chinese cultural context of a family-centric decision-making model, where the family serves as the core unit. Effective family communication and interaction patterns can reduce psychological resistance and cultural taboos surrounding discussions on death-related topics. Consequently, individuals not only feel less concerned about potential family conflicts arising from creating a Living Will but also benefit from their children’s understanding and support, which significantly enhances their awareness and acceptance of the Living Will system. A harmonious family atmosphere helps frame the act of creating a Living Will as a concrete manifestation of fulfilling family responsibilities, while stable, positive intergenerational relationships establish a solid familial foundation for promoting the acceptance of Living Wills.

#### 4.3.3 Middle-aged and older adults with a higher level of social support demonstrate a greater willingness to establish a living will

The study found that the coefficient for perceived subjective support among middle-aged and older adults was 0.039, with an odds ratio (*OR*) of 1.040 (95% CI: 1.010–1.071; *p* = 0.009). For each one-unit increase in subjective support, the odds of expressing willingness increased by 4.0%, indicating a statistically significant effect. For objective support, the coefficient was 0.025, with an *OR* of 1.025 (95% CI: 0.999–1.052; *p* = 0.055), suggesting a positive yet marginally significant influence on willingness (*p* = 0.055). Regarding the utilization of support, the coefficient was 0.031, with an *OR* of 1.031 (95% CI: 1.003–1.060; *p* = 0.027). Each one-unit increase in support utilization led to a 3.1% rise in the odds of willingness, demonstrating a significant effect. These findings are consistent with the results reported by Xu (18) and Rwabihama (19). When middle-aged and older adults can effectively access and utilize concrete social resources and support networks—such as policy consultation from communities, professional guidance from healthcare institutions, and document assistance from legal services—the core practical barrier of “not knowing how to proceed” in the process of understanding Living Will policies and preparing the relevant documents can be directly addressed. This, in turn, enhances their willingness to complete a Living Will.

### 4.4 Theory and empirical evidence

The findings of this study corroborate the Theory of Planned Behavior: “cognition” (knowledge of Living Wills), “attitude” (attitude toward Living Wills), and “subjective norms” (family support and social support) jointly influenced behavioral intention (willingness toward Living Wills). The results also affirm the Life Course Theory, demonstrating the dynamic process by which temporal factors (chronological age, generational cohort), hierarchical levels (individual psychological and physiological traits, socioeconomic status), and life domains (simultaneously experienced life spheres such as family, occupation, and education) exert a combined influence on the willingness toward Living Wills among middle-aged and older adults.

### 4.5 Study limitations

This study employed a cross-sectional survey, which revealed associations between willingness toward Living Wills and various factors but cannot establish causal relationships. The sample was predominantly recruited from healthcare service institutions, which may introduce systematic bias toward groups that are more health-conscious and have more frequent contact with the medical system. The identified associated factors are exploratory findings derived from the specific context of this study and may be constrained by the inherent limitations of the self-report measurement method (e.g., some individuals might overestimate their own health status or perceived support), potentially leading to inaccuracies in the research outcomes. For instance, the strong association observed between having witnessed medical rescue or end-of-life death and the willingness toward Living Wills, along with the substantially elevated effect size, might be influenced by self-report bias among participants, sample selection bias, and unmeasured confounding factors. Future research could conduct in-depth exploration across broader geographical regions. By expanding the coverage of the study areas, the external validity and generalizability of the findings across different cultural contexts and social backgrounds can be tested. Implementing dynamic follow-up and longitudinal cohort study designs, integrating multidisciplinary theoretical perspectives and diverse research methods to construct a more systematic and comprehensive analytical framework is recommended. This would provide a more targeted evidence base for policy formulation and practical intervention guidelines aimed at effectively enhancing the willingness toward Living Wills among middle-aged and older adults.

## 5 Summary

This study employed binary logistic regression analysis to systematically identify nine protective factors influencing the willingness toward Living Wills among adults aged 45 and above in Shenzhen. These factors include higher educational attainment, presence of religious belief, higher monthly income, experience of witnessing medical rescue or end-of-life death, better knowledge of Living Wills, a positive attitude toward Living Wills, good family function and psychological status, and stronger social support. Based on the empirical findings, we propose constructing a systematic intervention framework across multiple levels: implementing structured death education programs coupled with policy popularization on Living Wills, utilizing accessible formats like short videos and comics to enhance cognitive understanding and optimize attitudes; strengthening the mental health support service system to improve overall health status; promoting “family-style” interventions such as family workshops and parent–child communication guidance to enhance the quality of intra-family communication and emotional support; and simultaneously refining relevant supportive policies to reinforce the objective social support network. By leveraging a “community-healthcare-legal” collaborative network, these efforts can synergize with the cognitive advantages of highly literate groups, the planning foundation afforded by good health, and the emotional sustenance from strong family function, forming a “four-dimensional linkage” to collectively advance the transition of Living Wills from “willingness” to “practice.”

## Data Availability

The original contributions presented in the study are included in the article/supplementary material, further inquiries can be directed to the corresponding author.
